# Environmental Compensation Effect and Synergistic Mechanism of Optimized Nitrogen Management Increasing Nitrogen Use Efficiency in Indica Hybrid Rice

**DOI:** 10.3389/fpls.2019.00245

**Published:** 2019-03-04

**Authors:** Wei Zhou, Zhiping Yang, Tao Wang, Yong Fu, Yong Chen, Binhua Hu, Junko Yamagishi, Wanjun Ren

**Affiliations:** ^1^Key Laboratory of Crop Ecophysiology and Farming System in Southwest China, Ministry of Agriculture, Sichuan Agricultural University, Chengdu, China; ^2^Institute of Ecoagriculture, College of Agronomy, Sichuan Agricultural University, Chengdu, China; ^3^Graduate School of Agricultural and Life Sciences, The University of Tokyo, Bunkyo-ku, Japan; ^4^Institute of Biotechnology and Nuclear Technology, Sichuan Academy of Agricultural Sciences, Chengdu, China

**Keywords:** soil fertilizer, nitrogen utilization efficiency, *GS/GOGAT*, ^15^N-isotope, nitrogen absorption, nitrogen translocation

## Abstract

Modern rice cultivation relies heavily upon inorganic nitrogen fertilization. Effective fertilizer management is key to sustainable agricultural development. Field and pot trials were conducted in 2014–2016, including a ^15^N-labeled urea pot experiment (2014) to investigate mechanism by which optimized nitrogen fertilizer application (OFA) increases nitrogen utilization efficiency (NUE). Results showed that the applied nitrogen recovery efficiencies with OFA were 71.71%, 110.17%, and 51.38% higher than those obtained with traditional nitrogen fertilizer application (TFA) in 2014, 2015, and 2016, respectively. These improvements are attributed mainly to the high recovery efficiency rates derived from spikelet-developing and spikelet-promoting fertilizer applications at the jointing stage and 15–20 d after jointing. Under OFA, the amount of nitrogen fertilizer applied at the early stages was half that used in TFA, which not only promoted the absorption of soil nitrogen, but also reduced nitrogen loss to the environment, as the NUE of basal and tillering fertilizer was only about 22%. Nitrogen applied during the panicle differentiation stage increased the expression of *ATM1;1*, a NH_4_^+^ transporter in roots. This effect significantly improved the uptake of nitrogen derived from fertilizer from jointing to heading stage. Up-regulation of the expression and activity of *GS* and *GOGAT* at the panicle differentiation and grain-filling stages promoted nitrogen translocation from vegetative organs to reproductive organs. The uptake of nitrogen derived from fertilizer increased from 22.51% in TFA to 35.58% in OFA. Nevertheless, rice absorbs most of the nitrogen it requires from the soil. The OFA treatment could effectively utilize the environmental compensation effect, promote the absorption and transport of nitrogen, and ultimately lead to improvement in NUE. Future research should aim to understand the soil nitrogen supply capacity in order to apply nitrogenous fertilizer in such a way that it sustains the nitrogen balance.

## Introduction

Rice is the staple food for more than 65% of the Chinese and for 3 billion people worldwide (Zhang et al., 2005; Liu et al., 2016). According to the Food and Agriculture Organization of the United Nations^1^, the area under rice production and harvest in China was 19.1% and 28.5% of global production in 2016, respectively. Therefore, rice production in China affects food security in the country and even globally. Although rice yield per unit area in China was 1.5 times that of the world average (FAOSTAT, 2016), which attributable to a rapid increase in use of chemical nitrogen fertilizer. Furthermore, a close relationship was found between annual food production and utilization of nitrogen fertilizer (Zhu and Chen, 2002). Besides, under the unique conditions of paddy farming, nitrogen fertilizers can be easily transformed into NO_3_^-^ and NH_4_^+^ after fertilization and then lost to the environment through ammonia volatilization, leaching, denitrification, etc., which could cause many environmental pollution issues (Zhou et al., 2016b). For example, Erisman et al. (2007) calculated that the global ammonia emission from fertilizers every year was as much as 9.0 Tg N. About 15% of total nitrogen fertilizer used in agriculture is applied to rice alone (Heffer, 2009; Patil et al., 2010); it is therefore important in the context of sustainable development to improve nitrogen utilization rate (NUE) while ensuring rice yield.

China is currently the world’s largest consumer of nitrogen fertilizers, but the NUE has been as low as 35%—15%-20% lower than the other major rice production countries (Cao et al., 2013). High grain yield can be ensured at lower environmental cost by optimizing management strategies to meet the nitrogen demands of plants. Nitrogen absorption in rice varies with developmental stage. Nitrogen demand is greatest in the middle of the rice growth period, and can be as high as 9–12 kg N ha^-1^ d^-1^ (Peng and Cassman, 1998). At this time, panicle differentiation and functional leaf growth occur, and they determine grain yield potential. Therefore, nitrogen applied in the middle growth phase can promote plant development and nitrogen absorption, and substantially improve grain yield and NUE (Dobermann et al., 2002; Kamiji et al., 2011; Jia et al., 2016). Rice growth and development processes differ among rice varieties. Macroscopic observation alone does not suffice to determine critical fertilization timing. Extensive rice production experience has shown that panicle differentiation and intermodal elongation are almost isochronous in mid-season indica hybrid rice varieties, which are cultivated over a wide geographic range and play important roles in rice production in China. Therefore, we hypothesize that the application of topdressing at the jointing stage (nitrogen applied when the first internode of the base elongate about 2 cm) when the young panicle just start differentiating and 15–20 days after the jointing stage when the pollen mother cells undergo meiosis could meet the nitrogen demand of rice plants and improve rice yield and NUE.

Our previous studies have demonstrated the morphophysiological mechanisms of rice yield increase in response to this optimized nitrogen management (Zhou et al., 2017). The objective of the present study was to elucidate the mechanism by which optimized nitrogen management increases NUE. The roles of soil and fertilizer nitrogen in rice production in the Chengdu Plain (an important source of rice in China), and the importance of the topdressing at the jointing stage and 15–20 days after the jointing stage were also evaluated using the ^15^N tracer method. The findings of the present study could provide a theoretical basis for high-yield and high-efficiency production of other rice cultivars and promote sustainable development in agriculture.

## Materials and Methods

### Experimental Design

The experiments were conducted in the paddy fields of Wenjiang (30°43^′^ N and 103°52^′^ E), Sichuan, China, during the 2014–2016 rice growing seasons. The experimental soils were fluviatile sandy loams whose characteristics are described in Table 1. The rice cultivar Fyou498 (F32A × Shuhui498), a popular variety, was used as the test plant. The experimental design of the three years was showed in Table 2. The 2014 experiment was conducted as a pot trial with a single-factor, randomized block design. Three nitrogen treatments—traditional nitrogen fertilizer application (TFA; 70% basal and 30% tillering fertilizer; widely used by Chinese farmers), optimized nitrogen fertilizer application (OFA; 35% basal, 15% at early tillering, 25% at jointing, and 25% at 15–20 days after jointing), and no nitrogen application (control, CK)—were tested. The two nitrogen application regimes had the same total nitrogen amount of 1.2 g N pot^-1^, which was equivalent to a paddy application of 180 kg ha^-1^ at a density of 15 × 10^4^ hills ha^-1^. In addition, 10% ^15^N-labeled urea was applied as described in Supplementary Table 1. Phosphorus and potassium were applied to all treatments at 2.4 g P_2_O_5_ pot^-1^ and 1.2 g K_2_O pot^-1^, respectively. The height and diameter of the pot was 27 cm and 30 cm respectively; each pot was filled with 12.0 kg of air-dried, thoroughly mixed paddy topsoil. Each plot consisted of 22 pots with two plants in each pot, and all pots were placed in a greenhouse to prevent rainwater from leaching the applied fertilizers. The 2015 experiment was performed using a two-factor randomized block design with two nitrogen treatments and two nitrogen application rates. The nitrogen treatments were the same as in 2014, they were TFA, OFA, and CK. The nitrogen application rates were 180 kg ha^-1^ and 90 kg ha^-1^. In the 2016 field experiment, a single-factor, three-level (both TFA and OFA at 180 kg ha^-1^, and CK), randomized block design was conducted. In addition, 90 kg P_2_O_5_ ha^-1^ and 90 kg K_2_O ha^-1^was used in the base fertilizer, and 90 kg K_2_O ha^-1^ was used at the jointing stage in all treatments in the 2015 and 2016 field experiments. All fertilizers used in field experiments were conventional fertilizers. All treatments in field and pot experiments were repeated in triplicate. Seeds were sown on April 18 and transplanted on May 28–31 in the three years. The transplanting density was 15 × 10^4^ hills ha^-1^ (20 cm × 33 cm, two plants per hill). The area of the plot in the field was 12 m^2^ (3 m × 4 m). Ridges 20 cm high were built between plots and coated with plastic film to prevent fertilizer and water leaching into adjacent plots. Water management, pest- and disease control, and other rice management practices were similar for both the pot and the paddy trials, as described previously (Zhou et al., 2016a).

**Table 1:** Experimental soil characteristics.

Year	pH	Organic matter(g kg^-1^)	Total N(g kg^-1^)	Total P(g kg^-1^)	Total K(g kg^-1^)	Alkali-hydrolyzable N (mg kg^-1^)	Available P(mg kg^-1^)	Available K (mg kg^-1^)
2014	7.26	29.17	1.58	0.38	3.91	116.51	13.02	65.04
2015	5.80	29.58	1.28	0.50	7.75	97.64	20.27	56.52
2016	5.70	30.74	1.27	0.54	8.85	100.75	26.39	28.97


**Table 2:** The experimental design of the three years.

Year	Nitrogen dose (kg ha^-1^)	Nitrogen application	Experimental condition	Nitrogen form
2014	0,180^∗^	TFA,OFA	Pot	^15^N
2015	0,90,180	TFA,OFA	Field	^14^N
2016	0,180	TFA,OFA	Field	^14^N


*^∗^The nitrogen applied in pot experiment was 1.2 g N pot^-*1*^, which was equivalent to a paddy application of 180 kg ha^-*1*^ at a density of 15 × 10^*4*^ hills ha^-*1*^. TFA: traditional nitrogen fertilizer application, OFA: optimized nitrogen fertilizer application.*

### Indexes and Measurement Methods

#### Nitrogen Content and ^15^N Abundance Value

Six hills (two hills per plot with three replicates) with average numbers of tillers (determined from 60 hills of each treatment) were sampled from each treatment at the jointing, heading, and maturing stages in 2015 and 2016. Six pots with ^15^N-labeled urea applied were sampled at different stages, showed in Supplementary Table 1. All samples were divided into three parts (leaf, stem-sheath, and panicle), heated to 105 °C for 60 min, dried at 75 °C to a constant weight, and analyzed for dry matter content. After harvest, surface soils (0–20 cm depth) were sampled in triplicate from each plot within each treatment using an auger in 2014 and 2015. The soil samples were air-dried. The nitrogen contents (%) of all plant organs and soil samples were determined using the Kjeldahl method (Lu, 1999). The ^15^N abundance values were determined at the Shanghai Research Institute of Chemical Industry using mass spectrometry (Du et al., 2009). The soil total nitrogen content gradient was calculated from the difference between the soil total nitrogen content before transplantation of plants and after rice harvest divided by the soil total nitrogen content before transplantation.

#### Gene Expression and Enzyme Activity

Fresh white roots about 2cm length from the root tip and the middle of the newest fully extended leaves with the main vein stripped were sampled from 15 hills (five hills per plot with three replicates) to determine gene expression. Thirty new, fully-extended leaves with the main vein stripped (10 hills per plot with three replicates) from each treatment collected to detect enzyme activity. Samples were taken at jointing stage (T1), 15 d after jointing stage (immediately before fertilization, T2), at heading stage (T3), and 15 d after heading stage (T4); and were treated with liquid nitrogen immediately after collection and stored at -80 °C until use. RNA extraction and quantitative RT-PCR were conducted as described by Li et al. (2010), each biological replicate had two technical replicates. The primers for the qRT-PCR analysis of *AMT1;1, AMT1;2, AMT1;3, GS1;1, GS1;2, GS1;3, GS2, NADH-GOGAT1, NADH-GOGAT2*, and *Fd-GOGAT* expression are presented in Table 3. *GS* and *GOGAT* activities were measured using microdetermination kits (Suzhou Comin Biotechnology Co., Ltd., Shuzhou, Jiangshu, China) following the manufacturer’s protocol.

**Table 3:** Primers used in quantitative RT-PCR.

Genes	Forward primer	Reverse primer
*GS1;1*	CACCAACAAGAGGCACAATG	ACTCCCACTGTCCTGGCAT
*GS1;2*	TGTTTCTCCTCATCCCTGC	TCACAGTCCTCGCTTTGC
*GS1;3*	AGCCGATTCCGACGAACAAC	GTAGCGTGCCACCCAGACAT
*GS2*	ACCAAGAGTATGCGTGAAGA	AACCTGTCAACCTCCTTTCA
*AMT1;1*	GGTTTCTCTCCCTCTCCGAT	CCACCTTCACACCACACATT
*AMT1;2*	AAGCACATGCCGCAGACA	GACGCCCGACTTGAACAG
*AMT1;3*	GAACGCGACGGACTACC	CTGTGGGACCTGCTTGAG
*Fd-GOGAT*	GCATACTTGTGAAGCACCGAAGTG	CTGCAAATAGCAACCTAGCGTCAG
*NADH-GOGAT1*	GTGCAGCCTGTTGCAGCATAAA	CGGCATTTCACCATGCAAATC
*NADH-GOGAT2*	CCTGTCGAAGGATGATGAAGGTGAAACC	TGCATGGCCCTACTATCTTCGCATCA


#### Determination of Rice Yield

Rice was harvested at maturity and the yield of each plot recorded. After measuring the moisture content and removing the impurities, the standard grain output of each pot was determined based on a 13.5% moisture content.

#### Definition of Relative Parameters of NUE

The amount of nitrogen uptake was calculated by multiplying the productivity of different plant parts by their nitrogen content (%). The NUE calculation by the conventional difference method, including recovery efficiency (RE, express the rate of fertilizer N recovered in rice season), agronomic efficiency (defined as the increase in rice yield per kg N applied), physiological efficiency (the ratio of the increase in rice yield to the increase in nitrogen uptake), nitrogen harvest index (reflect the proportion of N in rice grain), partial factor production (the ratio of rice yield to N applied), nitrogen grain production efficiency (the ratio of rice yield to N uptake), and soil nitrogen dependent rate (SNDR, reflect the contribution of soil N to plant N nutrition) was performed as previously described (Zhou et al., 2016a):

RE (%)=(amount of N uptake in OFA or TFA−amount of N uptake in CK)/amount of N applied×100;

Agronomic efficiency (kg/kg)=(grain yield in OFA or TFA− grain yield in CK) amount of N applied;

Physiology efficiency (kg/kg)=(grain yield in OFA or TFA− grain yield in CK)/( amount of N uptake in OFA or TFA−amount of N uptake in CK);

N harvest index=amount of grain N uptake/total amount of plant N uptake;

Partial factor production=grain yield in OFA or TFA/amount of N applied;

N grain production efficiency (kg/kg)=grain yield per unit area/amount of plant N uptake per unit area;

SNDR (%)=total amount of plant N uptake in CK/total amount of plant N uptake in OFA or TFA×100.

The NUE determination using the ^15^N tracing method was conducted as described in Beeman and Arulmozhiselvan (2017). Nitrogen derived from the environment was calculated based on the difference between the total nitrogen accumulated in plants and nitrogen derived from fertilizer. The distribution and translocation of nitrogen were calculated as follows:

Distribution rate of leaves or stems (%)=amount of nitrogen accumulation in leaves or stems/total amount of nitrogen accumulation×100;

$$\mathrm{Translocation}\;\mathrm{amount}\;\mathrm{in}\;\mathrm{leaves}\;\mathrm{or}\;\mathrm{stems}\;(\mathrm{kg}\;{\mathrm{ha}}^{-1})=\mathrm{amount}\;\mathrm{of}\;\mathrm{nitrogen}\;\mathrm{accumulation}\;\mathrm{at}\;\mathrm{heading}\;\mathrm{stage}-\mathrm{amount}\;\mathrm{of}\;\mathrm{nitrogen}\;\mathrm{accumulation}\;\mathrm{at}\;\mathrm{maturing}\;\mathrm{stage};$$

Translocation rate of leaves or stems (%)=translocation amount of leaves or stems/amount of nitrogen accumulation in leaves or stems at heading stage×100.

#### Data Analysis

The data were subjected to ANOVA, with separate comparisons of treatments for each experiment, and a mapping analysis. Bar and line diagrams were used to assess the differences and tendencies among the means of different attributes. Comparisons were made using least significant difference (LSD) multiple range tests at the 0.01 and 0.05 significance levels.

## Results

### Increase in Nitrogen Utilization Efficiency

**FIGURE 1:**
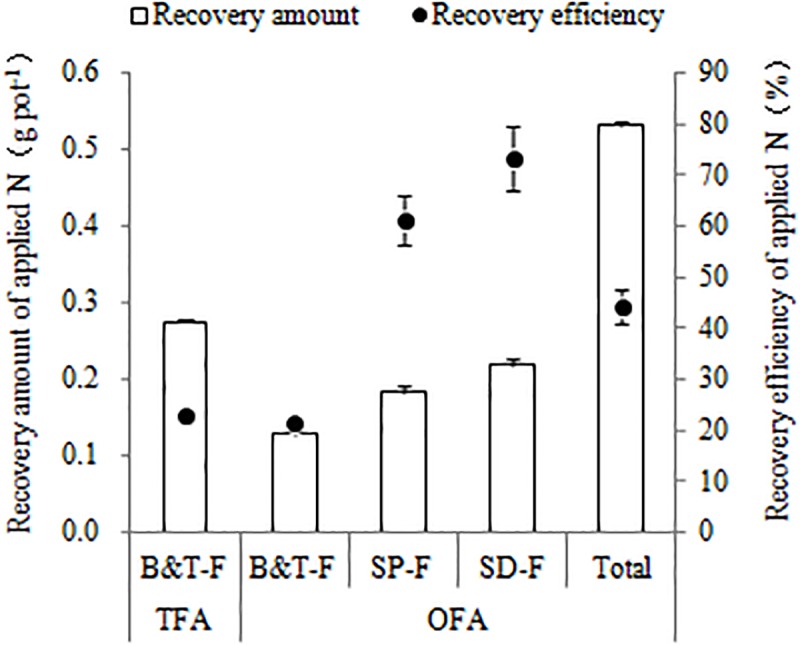
Quantity and efficiency of applied nitrogen recovery (2014, ^15^N tracing). TFA: traditional nitrogen fertilizer application, OFA: optimized nitrogen fertilizer application. B&T-F: base and tillering fertilizers; SP-F: spikelet-promoting fertilizer applied at jointing stage; SD-F: spikelet-developing fertilizer applied at 15–20 days after jointing stage. Data represent mean values of three biological replicates ± SE.

Differences between TFA and OFA in terms of the NUE-related parameters were consistent over the three years of the study. In OFA, nitrogen harvest index, partial factor production, agronomic efficiency, and RE all increased and nitrogen grain production efficiency, physiological efficiency, and SNDR decreased relative to those in TFA (Table 4). In 2016, the agronomic efficiency and partial factor production were 41.33% and 12.42% higher in OFA than in TFA, respectively. In the other testing years, there were no significant differences between OFA and TFA in terms of nitrogen harvest index, agronomic efficiency, physiological efficiency, and partial factor production. The RE of applied nitrogen is an important index of applied paddy nitrogen fertilizer utilization, which was 71.71%, 110.17%, and 51.38% higher in OFA than that in TFA in 2014, 2015, and 2016, respectively. The results of ^15^N tracing in 2014 also showed that the RE of OFA was 95.52% higher than that of TFA, but compared with the conventional difference method, the RE values reduced by 4.59% and 3.10%, respectively (Figure 1). The RE of the applied ^15^N fertilizer was 22.98% and 44.24% in TFA and OFA, respectively. Therefore, a proportion of the nitrogen absorbed by the rice was derived from an exchange between soil and fertilizer N. Under OFA conditions, this exchange capacity decreased. SNDR is defined as the ratio of the total nitrogen uptake in the absence of applied nitrogen to the total nitrogen uptake with nitrogen application. SNDR was lower in OFA than in TFA. Therefore, under the OFA conditions, more nitrogen fertilizer was assimilated by the plants and less was lost to the environment than under the TFA condition.

**Table 4:** Nitrogen utilization efficiency in the various treatments.

Year	Treatments	NHI	PFP	NGPE (kg kg^-1^)	AE^∗^ (kg kg^-1^)	PE ^∗^ (kg kg^-1^)	SNDR (%)	RE ^∗^ (%)
2014	TFA180	0.76	61.69	56.32	12.14	44.06	74.88a	27.57b
	OFA180	0.77	67.63	52.51	18.08	38.04	63.65b	47.34a
	F value	1.54ns	2.30ns	1.31ns	2.32ns	0.40ns	15.28^**^	33.83^**^
2015	TFA90	0.72a	107.75a	64.41a	12.85	42.95	81.79a	30.98b
	OFA90	0.73a	114.52a	54.60ab	19.62	28.99	65.67b	72.86a
	TFA180	0.67b	59.76b	58.77ab	12.31	38.07	67.29b	33.52b
	OFA180	0.68b	65.41b	49.84b	17.96	28.26	52.38c	62.69a
	TFA	0.70	83.76	61.59a	12.58	40.51	74.54a	32.25b
	OFA	0.71	89.97	52.22b	18.79	28.62	59.03b	67.78a
	F value	1.73ns	5.87ns	6.54^*^	4.39ns	1.41ns	29.60^**^	20.25^**^
	N90	0.73a	111.14a	59.51	16.23	35.97	73.73a	51.92
	N180	0.67b	62.58b	54.31	15.13	33.16	59.83b	48.11
	F value	24.81^**^	358.73^**^	1.96ns	0.14ns	0.09ns	24.13^**^	0.36ns
2016	TFA180	0.70	59.12b	55.53	17.76b	38.13	55.85	47.04
	OFA180	0.70	66.46a	51.18	25.10a	36.22	45.93	71.21
	F value	0.52ns	44.57^*^	1.44ns	44.57^*^	0.23ns	6.91ns	5.19ns


*^∗^These parameters were standardized for the control (CK) values. TFA: traditional nitrogen fertilizer application; OFA: optimized nitrogen fertilizer application; 90: total nitrogen application rate was 90 kg ha^-*1*^; 180: total nitrogen application rate was 180 kg ha^-*1*.^; RE: recovery efficiency; AE: agronomic efficiency; PE: physiological efficiency; NHI: nitrogen harvest index; PFP: partial factor production for applied nitrogen; NGPE: nitrogen grain production efficiency; SNDR: soil nitrogen dependent rate. Data represent mean values of three biological replicates, different lowercase letters beside numbers within the same column indicate significant differences at the 0.05 probability level according to the least significant difference (LSD) multiple-range test. “^∗^”, and “^∗∗^” indicate significant differences between treatments at the 0.05 and 0.01 probability levels, respectively; “ns”: not significant at the 0.05 probability level.*

^15^N tracing in 2014 indicated that the RE for basal and tillering fertilization was essentially the same in both OFA and TFA (22.98% and 21.33%, respectively; Figure 1). The recovery amount of applied nitrogen was highest when spikelet-developing fertilizer was applied at 15-20 d after jointing, followed by that for spikelet-promoting fertilizer applied at jointing, which was 0.22 and 0.18 g pot^-1^ respectively. This recovery rate was higher than those obtained for the basal and tillering fertilizer applications even though the latter two were applied at twice the rate of the former two. The RE for spikelet-developing and spikelet-promoting fertilizer was 73.17% and 61.15%, respectively, and they account for most of the increase in RE observed with OFA. After jointing, rice plants demand more nitrogen to meet their requirements for growth and development. Fertilization at the panicle differentiation stage promotes reproductive growth which, in turn, accelerates the absorption and utilization of applied nitrogen.

### Distribution and Source of Nitrogen Accumulated

**FIGURE 2:**
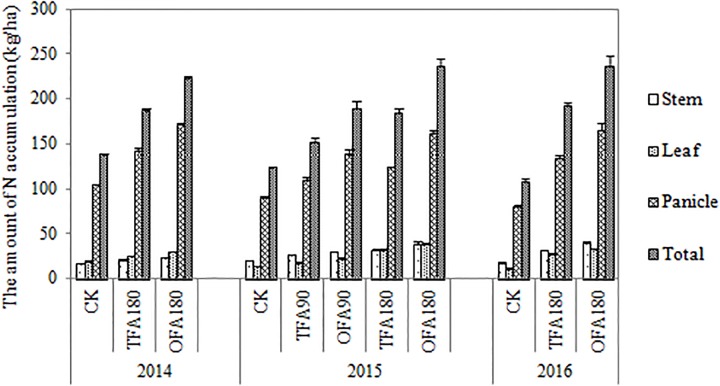
Nitrogen accumulation in the various treatments. CK: No nitrogen applied; TFA: traditional nitrogen fertilizer application; OFA: optimized nitrogen fertilizer application; 90: total nitrogen application rate of 90 kg ha^-1^; 180: total nitrogen application rate of 180 kg ha^-1^. Data represent mean values of three biological replicates ± SE.

**FIGURE 3:**
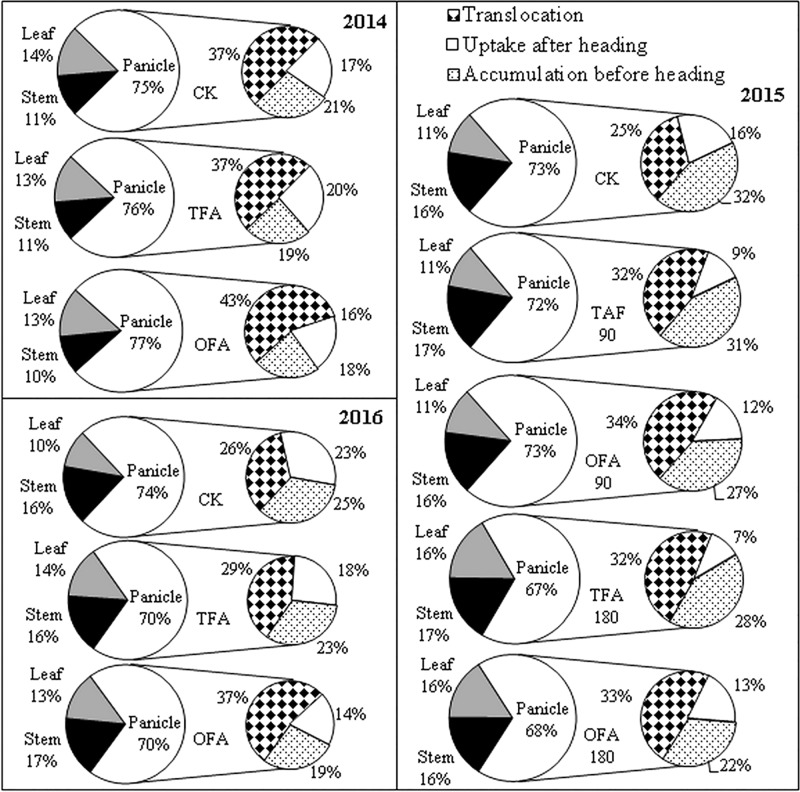
Nitrogen distribution and source accumulated in panicle. CK: No nitrogen applied, TFA: traditional nitrogen fertilizer application, OFA: optimized nitrogen fertilizer application, 90: total nitrogen application rate of 90 kg ha^-1^; 180: total nitrogen application rate of 180 kg ha^-1^. Data represent mean values of three biological replicates.

The relatively high RE of OFA indicated greater nitrogen accumulation at the mature stage. Figure 2 shows the differences in total nitrogen uptake among the various nitrogen applications. The amount of nitrogen accumulated increased with nitrogen application rate. The nitrogen content in the panicles was significantly higher than in the leaves and stems. The nitrogen accumulation in the leaves, stems, and panicles of rice treated with OFA was greater than that in rice treated with TFA, which on average increased by 18.70%, 22.07%, and 25.33% of the three years, respectively. These increases in different organs contributed to the 23.82% increment in total nitrogen uptake by the rice plants in OFA. After heading, nitrogen was transported from nutritive organs to panicles for grain development; at the mature stage only 10%-16% and 10%-17% nitrogen stayed in the leaves and steams, respectively, and most of the nitrogen accumulated in the panicles (Figure 3). Therefore, increasing the panicle nitrogen content is key to increase overall plant nitrogen absorption and utilization.

**FIGURE 4:**
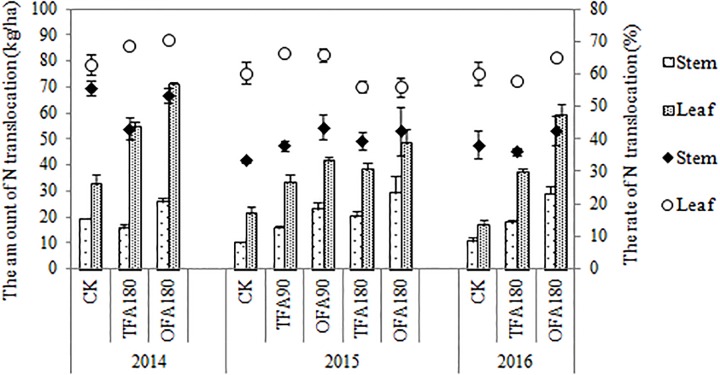
Differences in the quantities and rates of nitrogen translocation. CK: no nitrogen applied; TFA: traditional nitrogen fertilizer application; OFA: optimized nitrogen fertilizer application; 90: total nitrogen application rate of 90 kg ha^-1^; 180: total nitrogen application rate of 180 kg ha^-1^. Data represent mean values of three biological replicates ± SE.

Nitrogen accumulation in the panicle is derived from foliar and stem translocation, uptake after heading, and accumulation in the panicle during differentiation. Results for the three years of the study indicated that most of the nitrogen in the panicle originated from translocation from the leaves and stems, especially for the nitrogen application treatments, for which it could be as high as about 50%, while the proportion of nitrogen absorbed after heading could be as least as 7%. OFA promoted this translocation (Figure 3). The amount and rate of nitrogen translocation from the leaves were greater than those from the stems (Figure 4). The quantities of nitrogen translocated from the leaves in OFA were 29.61%, 25.82%, and 58.04% greater than those in TFA in 2014, 2015, and 2016, respectively. The amounts of nitrogen translocated from the stems in OFA were 66.45%, 46.23%, and 62.19% larger than those in TFA in the three respective experimental years. In contrast, no significant differences were found between OFA and TFA in terms of nitrogen translocation rate. In fact, the foliar translocation rate in OFA was slightly lower than that in TFA. Therefore, to some extent, both OFA and TFA can potentially improve NUE by promoting nitrogen translocation from the vegetative to the reproductive organ during the grain-filling stage.

### Nitrogen Derived From the Environment

**FIGURE 5:**
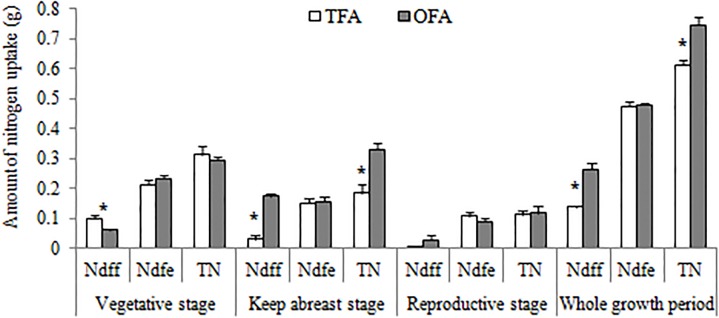
Nitrogen derived from fertilizer and environment (2014, ^15^N tracing). TFA: Traditional nitrogen fertilizer application; OFA: optimized nitrogen fertilizer application; Ndff: nitrogen derived from fertilizer; Ndfe: nitrogen derived from the environment; TN: total nitrogen uptake; Data represent mean values of three biological replicates ± SE. “^∗^” indicates significant difference between treatments at the 0.05 probability level.

Table 4 shows the reduction of SNDR in OFA. Nevertheless, the SNDR still reached 46%–66% in OFA and 56%–82% in TFA. Therefore, soil fertility significantly contributes to rice production during the paddy season. The ^15^N tracing test in 2014 also demonstrated the importance of nitrogen derived from the environment for rice growth (Figure 5). The amount of environmental nitrogen uptake decreased as the rice plants grew. The contributions of nitrogen derived from the environment were 67.79%, 82.57%, and 96.10% in TFA during the vegetative, keep-abreast, and reproductive stages, respectively. The contributions of environmentally derived nitrogen were 79.12%, 47.36%, and 75.37% in OFA during the same aforementioned growth stages. Except for the keep-abreast stage in OFA, these values were significantly higher than those obtained for fertilizer nitrogen. Only half the amount of nitrogen applied in TFA during the vegetative stage was used in OFA. Therefore, relative to TFA, less nitrogen was derived from fertilizer and more from the environment in OFA. In contrast, very little nitrogen was derived from fertilizer in the reproductive stage under TFA because the fertilizers were applied only at the earlier growth stages. For this reason, relatively more nitrogen was derived from the environment during the reproductive stage in TFA than in OFA. The failure of fertilizer to meet plant nitrogen requirements would promote soil nitrogen uptake.

**FIGURE 6:**
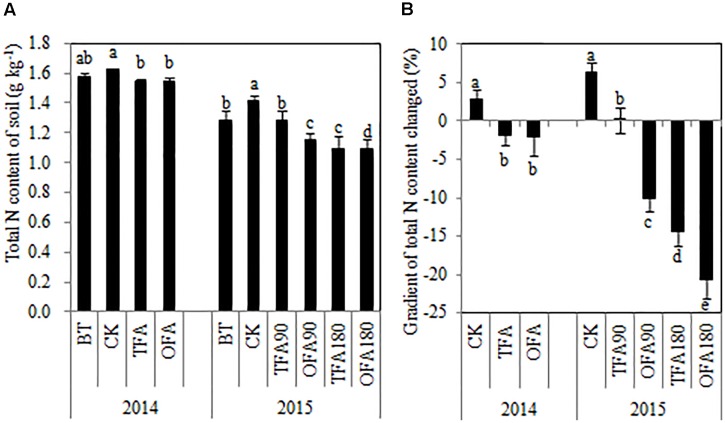
Changes in paddy soil nitrogen after application of different nitrogen sources. **(A)** Total nitrogen content of soil; **(B)** gradient of total soil nitrogen content changed. BT: before transplanting, CK: no nitrogen applied, TFA: traditional nitrogen fertilizer application, OFA: optimized nitrogen fertilizer application, 90: total nitrogen applied rate was 90 kg ha^-1^, 180: total nitrogen applied rate was 180 kg ha^-1^. Data represent mean values of three biological replicates ± SE. The different lowercase letters indicate significant difference at 0.05 probability level according to LSD multiple-range test.

As shown in Figure 6, the soil total nitrogen content actually increased after one paddy season in CK treatments. In contrast, except for TFA90, all of treatments in which fertilizer nitrogen was applied reduced the soil total nitrogen content relative to that measured before rice transplantation. The total nitrogen accumulated by the rice plants at the mature stage (Figure 2) varied inversely with soil total nitrogen content (Figure 6). Therefore, in OFA, the SNDR decreased but the total amount of nitrogen derived from the soil increased along with the plant nitrogen requirement. In general, the improvement in NUE was the result of increases in the amount of fertilizer nitrogen absorbed by the plants, especially in the keep-abreast stage. At that time in OFA, 65.71% of the total nitrogen was derived from fertilizer, and this quantity was 440.82% higher than that of TFA.

### Promotion of Nitrogen Absorption and Transport

**FIGURE 7:**
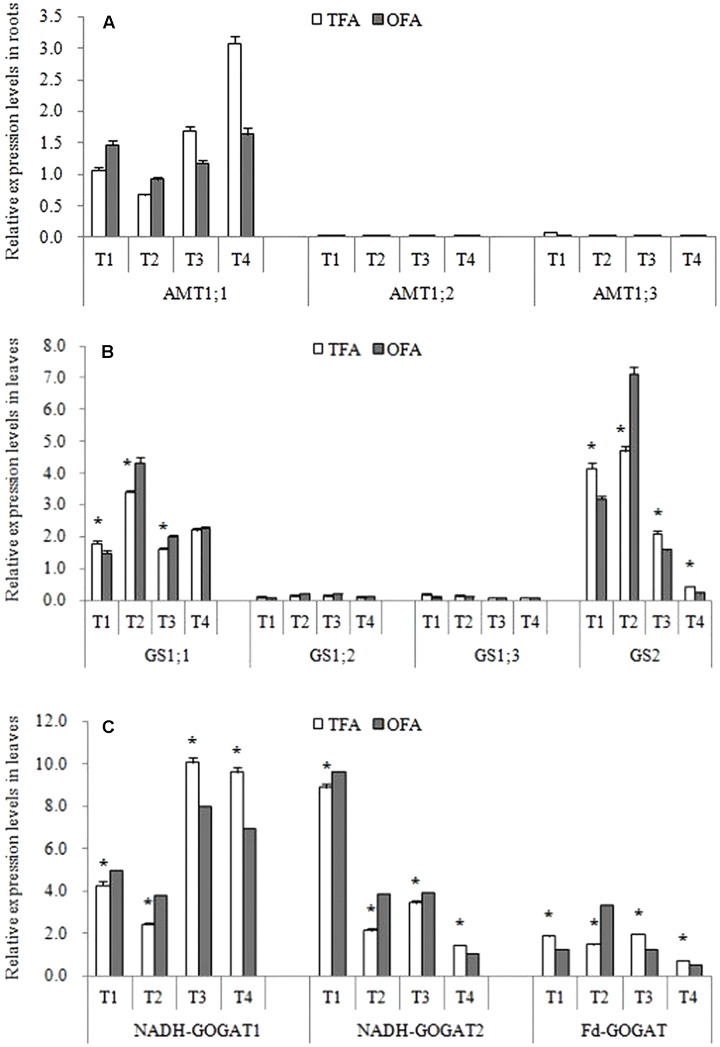
Relative gene expression levels under various nitrogen source treatments (2016). **(A)** Relative expression levels of AMT in roots; **(B)** relative expression levels of GS in leaves; **(C)** relative expression levels of GOGAT in leaves. TFA: Traditional nitrogen fertilizer application; OFA: optimized nitrogen fertilizer application; T1: jointing stage; T2: 15–20 days after jointing; T3: heading stage; T4: 15 days after heading stage. Data represent mean values of three biological replicates (each bioogical replicate had two technical replicates) ± SE. “^∗^” indicate significant difference between treatments at the 0.05 probability level.

Figure 5 shows that the differences in total plant nitrogen accumulation were caused mainly by nitrogen uptake during the keep-abreast stage when the panicle fertilizer was applied. *AMT* family genes are the key gene for ammonium uptake in rice roots. Results showed that *AMT1;2* and *AMT1;3* were only slightly expressed in the roots after jointing (Figure 7A). In the panicle differentiation stages (T1 and T2), however, the expression levels of *AMT1;1* were higher in OFA than in TFA. In contrast, *AMT1;1* expression was higher after heading in TFA than in OFA. The relatively higher *AMT1;1* expression at panicle differentiation in OFA may have been induced by fertilizer application. The comparatively higher *AMT1;1* expression in TFA post-heading may be explained by an increase in soil uptake by the roots in the attempt to meet the nitrogen demand for grain filling. This finding may account for the relative increase in environmentally derived nitrogen in the reproductive-stage plants under TFA (Figure 5).

**FIGURE 8:**
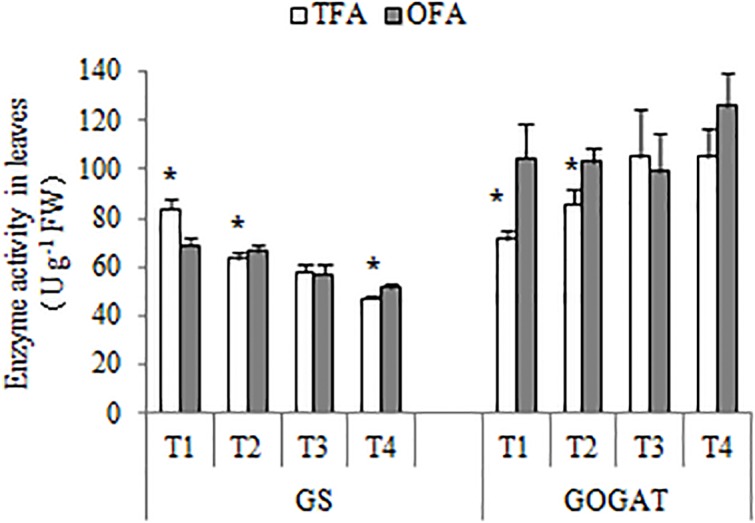
*GS* and *GOGAT* activities under various nitrogen source treatments (2016). TFA: traditional nitrogen fertilizer application; OFA: optimized nitrogen fertilizer application; T1: jointing stage; T2: 15–20 days after jointing; T3: heading stage; T4: 15 days after heading; FW: fresh weight. Data represent mean values of three biological replicates ± SE. “^∗^” indicates significant differences between treatments at the 0.05 probability level.

About half of the nitrogen accumulated in the panicles originated from foliar translocation (Figure 3, 4). The *GS/GOGAT* cycle plays an important role in nitrogen remobilization and translocation in rice leaf during grain filling stage. Its activity and expression level are influenced by several factors. Except for the minimal expression of *GS1;2* and *GS1;3* after jointing, all other gene expression levels and activities related to *GS* and *GOGAT* were significantly up-regulated at T2 after fertilization during panicle differentiation in OFA. After heading, except for *GS1;1* and *NADH-GOGA*T2 in T3, the expression levels of all other *GS-* and *GOGAT-*related genes were lower in OFA than in TFA (Figure 7B,C). The activities of *GS* and *GOGAT* were not synchronized with their expression levels (Figure 8). At T4, the activities of *GS* and *GOGAT* were higher in OFA than in TFA. Earlier studies showed that OFA delayed leaf senescence possibly by reducing nitrogen translocation between T3 and T4. Nevertheless, the total nitrogen translocation from the vegetative organs was greater in OFA than in TFA (Figure 3, 4). Thereafter, nitrogen translocation should accelerate, resulting in lower and higher enzyme activities of OFA at T3 and T4, respectively. The incongruence between gene expression and enzyme activity data had been also found in other studies, which maybe related to the multilevel regulation of GS (Hanne et al., 2014).

## Discussion

### Environmental Compensation Effect for Rice Growth

It is imperative to maintain and/or improve soil quality for crop production, as the total area of arable land has been decreasing and the population and food demand increasing steadily (Peng et al., 2009; Gathala et al., 2011). Nitrogen is a key factor for soil productivity and crop growth. The rice crop will probably be deficient in nitrogen when the soil total nitrogen content is < 0.2%. While, most of the arable land in China has < 0.2% N (1.27–1.58 g kg^-1^ in this study, Table 1), nearly all of it requires nitrogen fertilizer application. Although fertilization is an important source of soil nitrogen, it does not suffice to ensure high yields of hybrid rice (Huang et al., 2017). Studies on various wetland rice soils indicated that only 20%–30% of the total nitrogen uptake at crop maturity was derived from fertilizer, and the soil mineral nitrogen content before transplantation was significantly correlated with nitrogen uptake by plant (Schnier et al., 1987). The ^15^N tracing test in this study also showed that only 22.51% in TFA and 35.58% in OFA (Figure 5) of nitrogen absorbed by plants was derived from fertilizer. The higher SNDR from the three years’ results also corroborate this notion (Table 4). Plant nitrogen demand and soil nitrogen application efficacy codetermine the amount of nitrogen that the plants acquire from the soil/environment. Treatments with nitrogen compared with CK, and OFA treatments compared with TFA treatments, increased the plant nitrogen demand, and improved soil nitrogen uptake, which was defined by Hamid and Ahmad (1993) as the “priming effect” or “added nitrogen interaction.” This explains why soil nitrogen content after one rice season was lower for all nitrogen fertilizer treatments than it was for CK (Figure 6). The greater the plant nitrogen demand, the greater the soil nitrogen uptake—especially when fertilizer nitrogen cannot meet the demand of the plants. The increase of soil nitrogen uptake in vegetative stage under OFA conditions and in reproductive stage under TFA condition (Figure 5) was a good representation of this phenomenon. However, the amount of nitrogen in the plants derived from the soil decreased as rice growth progressed. Rice is often cultivated in rotation with other upland crops, which has significant effects on paddy soil nitrogen content. A wheat-rice rotation study revealed that after plowing and flooding for rice production, ∼69% of the mineral nitrogen accumulated after wheat harvest was lost in 13 d (Fan et al., 2007). Nitrogen applied to paddies promoted the loss of native soil nitrogen by denitrification, leaching, and NH_3_ volatilization (Zhao et al., 2016). The aforementioned processes, together with plant absorption of soil nitrogen, reduce the soil nitrogen supply capacity throughout the rice growth period.

The amount of nitrogen required by high-yield rice is substantial. Satoshi et al. (2013) reported that to achieve high grain yields (8 t ha^-1^ and 9 t ha^-1^ for japonica-dominant and indica-dominant varieties, respectively), an uptake of 15 g N m^-2^ is necessary. Even more nitrogen is required by indica hybrid rice varieties including Fyou498 because their yield potential is > 10 t ha^-1^ (Zhou et al., 2016a). In the present study, the preceding crop was wheat. Nevertheless, in Wenjiang, most of the land is planted with garlic because of its high commercial value, which may highlight the environmental nitrogen supply capacity, as Fan et al. (2008) reported that irrigation water and rainfall supplied as much as ∼5.3 kg N ha^-1^ and ∼8.3 kg N ha^-1^, respectively, in the Wenjiang rice-garlic planting area. Therefore, effective utilization of environmental nitrogen, especially in the vegetative stage of the rice plant when the soil nitrogen supply capacity is high and easily lost, and using effective fertilization management are of vital importance to maintain soil fertility and rice productivity.

### Synergistic Mechanism in Nitrogen Utilization

The nitrogen required for grain filling is acquired by direct soil absorption and by remobilization from the leaves and stems. Previous studies demonstrated that the nitrogen translocation efficiency ranged from 44.7% to 66.7% in various rice cultivars and the newly absorbed nitrogen accounted for only 10%–30% of the total in the panicles (Mae, 1997; Ntanos and Koutroubas, 2002). Data over the three years of the study also demonstrated the importance of nitrogen translocation. The genes/enzymes *GS/GOGAT* cycle plays a very important role in nitrogen remobilization (Yamaya and Kusano, 2014). The relative gene expression and enzyme activities associated with the *GS/GOGAT* cycle are affected by nitrogen fertilizer application but are not consistent through all of the rice growth stages (Figure 7, 8). When nitrogen fertilizer was applied in the panicle differentiation stage under OFA, the *GS* and *GOGAT* gene expression and enzyme activity levels were regulated by the nitrogen level (Zhao and Shi, 2006; Hanne et al., 2014). When newly absorbed nitrogen failed to meet the demand of panicle development, nitrogen translocation was promoted. The nitrogen supply during panicle development was greater in OFA than it was in TFA. Nevertheless, the nitrogen demand was also greater at this stage, as demonstrated in our previous study showing that OFA may increase the number of spikelets and improve rice production (Zhou et al., 2016a, 2017). *GS* and *GOGAT* also have other roles in grain yield, including the number of spikelets (Hanne et al., 2014; Yamaya and Kusano, 2014). Therefore, those results were obtained under the combined actions of nitrogen supply and demand. Nitrogen applied at panicle differentiation significantly affected T2. At that stage, the gene expression and enzyme activities of *GS* and *GOGAT* were higher in OFA than in TFA. Further studies should be conducted to determine *GS* and *GOGAT* regulation and balance in various plant development processes.

The OFA improves NUE because it involves correctly timed split dressing. The nitrogen fertilizer application rate in the base and early tillering stage in OFA was half that in TFA. Rice growth was not adversely affected, the proportion of ineffective tillering decreased, the rate of ear-bearing tillers increased, and the growth of superior tillers was promoted (Zhou et al., 2017). Excess nitrogen fertilizer may be rapidly lost to the environment in the form of ammonia volatilization, denitrification, and leaching (Zhou et al., 2016b). Soil nitrogen supply capacity was higher at the early stage which, together with nitrogen fertilizer application, provides enough nitrogen for plant growth. Rice plants can absorb nitrogen throughout their entire growth period, but the absorption peak occurs at panicle differentiation stage (Schnier et al., 1987; Peng and Cassman, 1998). The present study showed that in OFA, the rice plants accumulated the most nitrogen during the keep-abreast stage, and it was 79.03% higher than that measured under the TFA treatment. The plant nitrogen demand and the exogenous nitrogen application rates at jointing and 15–20 days after jointing up-regulated *AMT1;1*, which participates in NH_4_^+^ absorption and translocation (Von Wirén et al., 1997). Increased *AMT1;1* expression at that time promoted nitrogen uptake to accommodate the nutrient demands at the mid and late growth stages of indica hybrid rice. According to the results of this study, certain slow- or controlled-release fertilizers could be used in mid-season indica hybrid rice production if they can supply nutrients for ≥ 80 d and release most of the nutrients between 30 and 50 days.

**FIGURE 9:**
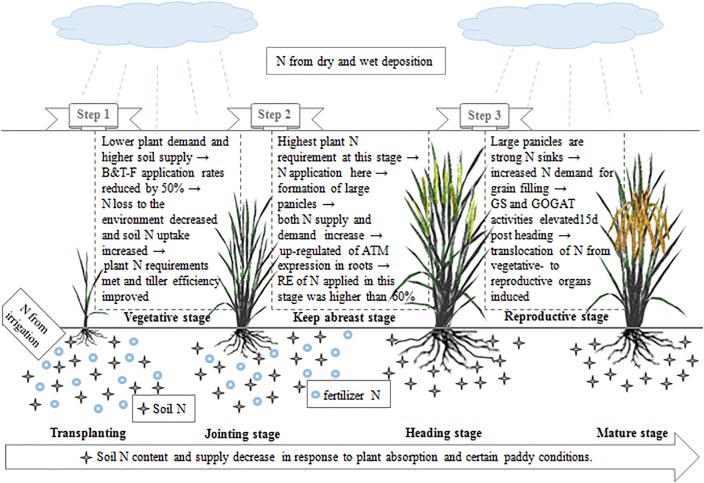
Mechanism by which nitrogen utilization efficiency is improved in indica hybrid rice in response to optimized nitrogen management.

From the above, we can conclude that nitrogen absorption and translocation are two important determinants of nitrogen utilization. The mechanism by which NUE increases in response to OFA in indica hybrid rice is shown in Figure 9. With OFA, the relatively low application rates during the early rice growth stages reduced fertilizer nitrogen loss to the environment, promoted soil nitrogen absorption, met plant nutrient demands, and reduced the formation of inefficient tillers. The expression of genes and the activity of enzymes related to nitrogen absorption (*AMT*), remobilization, and translocation (*GS* and *GOGAT*) were regulated by the nitrogen supply and demand. Nitrogen fertilizers applied at jointing and 15–20 days after jointing up-regulated *AMT* expression in the roots at panicle differentiation and increased *GS/GOGAT* activity at grain filling. In this way, fertilizer nitrogen absorption and transport from the vegetative organs to grains were promoted. Therefore, the nitrogen requirement at the mid and late stages of rice growth and development were met. Nitrogen derived from fertilizer increased from 22.51% in TFA to 35.58% in OFA. Consequently, OFA significantly improved the RE of applied nitrogen relative to that for TFA. Although the dependence on soil nitrogen remained relatively high with OFA, effective cultivation techniques and fertilization management will help maintain soil productivity.

## Author Contributions

WR and YC conceived the original screening and research plans. WZ, ZY, TW and YF performed most of the experiments. BH determined and analyzed the genes expression level. WZ designed the experiments, analyzed the data, and wrote the article with contributions of all the authors. WR and JY supervised and complemented the writing.

## Conflict of Interest Statement

The authors declare that the research was conducted in the absence of any commercial or financial relationships that could be construed as a potential conflict of interest.

## Footnotes

1 FAO, http://www.fao.org/faostat/en/#home.

## Abbreviations

CK: zero nitrogen application served as the control
NUE: nitrogen use efficiency
OFA: optimized nitrogen fertilizer application
RE: recovery efficiency
SNDR: soil nitrogen dependent rate
TFA: traditional nitrogen fertilizer application
