# Supplementary material for: Environmental Compensation Effect and Synergistic Mechanism of Optimized Nitrogen Management Increasing Nitrogen Use Efficiency in Indica Hybrid Rice
Source: Front Plant Sci. 2019 Mar 4;10:245. doi: 10.3389/fpls.2019.00245 (PMC6410729; doi:10.3389/fpls.2019.00245)
Supplement: Supplementary file 1 [file Table_1.DOCX]

Supplementary Table 1. Pots with ^15^N-labeled urea applied and the sampling stage.

| Treatments | B-F | T-F | SP-F | SD-F | No. of pots | Sampling stage |
| --- | --- | --- | --- | --- | --- | --- |
| CK | / | / | / | / | 30 | JS, HS, MS |
| TAF | common urea | common urea | / | / | 42 | / |
|  | ^15^N-labeled urea | ^15^N-labeled urea | / | / | 24 | JS, HS, MS |
|  |  |  |  |  |  |  |
| OFA | Common urea | Common urea | Common urea | Common urea | 18 | / |
|  | ^15^N-labeled urea | ^15^N-labeled urea | Common urea | Common urea | 18 | JS, MS |
|  | ^15^N-labeled urea | ^15^N-labeled urea | ^15^N-labeled urea | Common urea | 12 | MS |
|  | ^15^N-labeled urea | ^15^N-labeled urea | ^15^N-labeled urea | ^15^N-labeled urea | 18 | HS, MS |

CK: no nitrogen applied; TFA: traditional nitrogen fertilizer application, OFA: optimized nitrogen fertilizer application. B-F: base fertilizers; T-F: tillering fertilizers; SP-F: spikelet-promoting fertilizer applied at jointing stage; SD-F: spikelet-developing fertilizer applied at 15-20 d after jointing stage; JS: jointing stage; HS: heading stage; MS: maturing stage.
